# Extreme heterogeneity in sex chromosome differentiation and dosage compensation in livebearers

**DOI:** 10.1073/pnas.1905298116

**Published:** 2019-09-04

**Authors:** Iulia Darolti, Alison E. Wright, Benjamin A. Sandkam, Jake Morris, Natasha I. Bloch, Marta Farré, Rebecca C. Fuller, Godfrey R. Bourne, Denis M. Larkin, Felix Breden, Judith E. Mank

**Affiliations:** ^a^Department of Genetics, Evolution and Environment, University College London, London WC1E 6BT, United Kingdom;; ^b^Department of Animal and Plant Sciences, University of Sheffield, Sheffield S10 2TN, United Kingdom;; ^c^Department of Zoology, University of British Columbia, Vancouver, BC V6T 1Z4, Canada;; ^d^Department of Biomedical Engineering, University of Los Andes, Bogotá 111711, Colombia;; ^e^School of Biosciences, University of Kent, Canterbury CT2 7NJ, United Kingdom;; ^f^Department of Animal Biology, University of Illinois at Urbana–Champaign, Urbana, IL 61801;; ^g^Department of Biology, University of Missouri–St. Louis, St. Louis, MO 63105;; ^h^Department of Comparative Biomedical Sciences, Royal Veterinary College, London NW1 0TU, United Kingdom;; ^i^Department of Biological Science, Simon Fraser University, Burnaby, BC V5A 1S6, Canada;; ^j^Department of Organismal Biology, Uppsala University, Uppsala 752 36, Sweden

**Keywords:** Y degeneration, dosage compensation, recombination, poeciliids

## Abstract

Once recombination is halted between the X and Y chromosomes, sex chromosomes begin to differentiate and transition to heteromorphism. While there is a remarkable variation across clades in the degree of sex chromosome divergence, far less is known about the variation in sex chromosome differentiation within clades. Here, we combined whole-genome and transcriptome sequencing data to characterize the structure and conservation of sex chromosome systems across Poeciliidae, the livebearing clade that includes guppies. We found that the *Poecilia reticulata* XY system is much older than previously thought, being shared not only with its sister species, *Poecilia wingei*, but also with *Poecilia picta*, which diverged roughly 20 million years ago. Despite the shared ancestry, we uncovered an extreme heterogeneity across these species in the proportion of the sex chromosome with suppressed recombination, and the degree of Y chromosome decay. The sex chromosomes in *P. reticulata* and *P. wingei* are largely homomorphic, with recombination in the former persisting over a substantial fraction. However, the sex chromosomes in *P. picta* are completely nonrecombining and strikingly heteromorphic. Remarkably, the profound degradation of the ancestral Y chromosome in *P. picta* is counterbalanced by the evolution of functional chromosome-wide dosage compensation in this species, which has not been previously observed in teleost fish. Our results offer important insight into the initial stages of sex chromosome evolution and dosage compensation.

## Significance

Morphologically and functionally distinct X and Y chromosomes have repeatedly evolved across the tree of life. However, the extent of differentiation between the sex chromosomes varies substantially across species. As sex chromosomes diverge, the Y chromosome gene activity decays, leaving genes on the sex chromosomes reduced to a single functional copy in males. Mechanisms have evolved to compensate for this reduction in gene dosage. Here, we perform a comparative analysis of sex chromosome systems across poeciliid species and uncover extreme variation in the degree of sex chromosome differentiation and Y chromosome degeneration. Additionally, we find evidence for a case of chromosome-wide dosage compensation in fish. Our findings have important implications for sex chromosome evolution and regulation.

Sex chromosome evolution is characterized by remarkable variation across lineages in the degree of divergence between the X and Y chromosomes (1, 2). Derived from a pair of homologous autosomes, sex chromosomes begin to differentiate as recombination between them is suppressed in the heterogametic sex over the region spanning a newly acquired sex-determining locus (3, 4). The lack of recombination exposes the sex-limited Y chromosome to a range of degenerative processes that cause it to diverge in structure and function from the corresponding X chromosome, which still recombines in females (5, 6). Consequently, the sex chromosomes are expected to eventually transition from a homomorphic to heteromorphic structure, supported by evidence from many of the old and highly differentiated systems found in mammals (7, 8), birds (9), *Drosophila* (5), and snakes (10).

However, there is a significant heterogeneity among clades, and even among species with shared sex chromosome systems, in the spread of the nonrecombining region, and the subsequent degree of sex chromosome divergence (11–13). Age does not always reliably correlate with the extent of recombination suppression, as the sex chromosomes maintain a largely homomorphic structure over long evolutionary periods in some species (12, 14–17), while the 2 sex chromosomes are relatively young, yet profoundly distinct, in others (18). Comparing the structure and recombination patterns of sex chromosomes between closely related species is a powerful method to determine the forces shaping sex chromosome evolution over time.

Sex chromosome divergence can also lead to differences in X chromosome gene dose between males and females. Following recombination suppression, the Y chromosome undergoes gradual degradation of gene activity and content, leading to reduced gene dose in males (6, 19, 20). Genetic pathways that incorporate both autosomal and sex-linked genes are primarily affected by such imbalances in gene dose, with potential severe phenotypic consequences for the heterogametic sex (21). In some species, this process has led to the evolution of chromosome-level mechanisms to compensate for the difference in gene dose (22, 23). However, the majority of sex chromosome systems are associated with gene-by-gene level mechanisms, whereby dosage-sensitive genes are compensated, but overall expression of the X chromosome is lower in males compared with females (20, 23, 24).

As opposed to most mammals and birds, the sex chromosomes of many fish, lizard, and amphibian species are characterized by a lack of heteromorphism, which has usually been attributed to processes such as sex chromosome turnover and sex reversal (16, 25–30). As a result, closely related species from these taxonomic groups often have a variety of sex chromosome systems found at different stages in evolution (27, 31–33). Alternatively, undifferentiated sex chromosomes in anolis lizards, for example, have been found to be the result of long-term conservation of a homomorphic ancestral system (34). Additionally, global dosage compensation has not yet been found in fish, perhaps due to the transient nature of the sex chromosome systems and the general lack of heteromorphism in the group. However, incomplete dosage compensation, through a gene-by-gene regulation mechanism, may have evolved in sticklebacks (35, 36), flatfish (37), and rainbow trout (38).

Poeciliid species have been the focus of many studies concerning sex determination (26). Moreover, many poeciliids exhibit sexual dimorphism, with some color patterns and fin shapes controlled by sex-linked loci (39–43). The clade also has a diversity of genetic sex determination systems, with both male and female heterogametic sex chromosomes observed in different species (44, 45). Most work on poeciliid sex chromosome structure has focused on the *Poecilia reticulata* XY system, positioned on chromosome 12 (46), which shows very low levels of divergence (42, 47). Although recombination is suppressed over almost half the length of the *P. reticulata* sex chromosome, there is little sequence differentiation between the X and Y chromosomes and no perceptible loss of Y-linked gene activity in males (47). This low level of divergence suggests a recent origin of the sex chromosome system.

There is intraspecific variation in the extent of the nonrecombining region within *P. reticulata*, correlated with the strength of sexual conflict (47). Additionally, although *P. reticulata* and its sister species, *Poecilia wingei*, are thought to share an ancestral sex chromosome system (48, 49), there is some evidence for variation in Y chromosome divergence between these species (49). It is unclear whether the XY chromosomes maintain the same level of heteromorphism in other poeciliids (44, 48), or even whether they are homologous to the sex chromosomes in *P. reticulata*.

Here, we perform comparative genome and transcriptome analyses on multiple poeciliid species to test for conservation and turnover of sex chromosome systems and investigate patterns of sex chromosome differentiation in the clade. We find the XY system in *P. reticulata* to be older than previously thought, being shared with both *P. wingei* and *Poecilia picta*, and thus dating back to at least 20 million years ago (mya). Despite the shared ancestry, we uncover an extreme heterogeneity across these species in the size of the nonrecombining region, with the sex chromosomes being largely homomorphic in *P. reticulata* and *P. wingei*, while completely nonrecombining and highly diverged across the entire chromosome in *P. picta*. Remarkably, although the Y chromosome in *P. picta* shows signatures of profound sequence degeneration, we observe equal expression of X-linked genes in males and females, which we find to be the result of dosage compensation acting in this species. Chromosome-wide sex chromosome dosage compensation has not been previously reported in fish.

## Results and Discussion

### Comparative Assembly of Poeciliid Sex Chromosomes.

We sequenced the genome and transcriptome of 3 male and 3 female individuals from each of the 4 target species (*P. wingei*, *P. picta*, *Poecilia latipinna*, and *Gambusia holbrooki*) (*SI Appendix*, Table S1) chosen to represent an even taxonomic distribution across Poeciliidae. For each species, we generated DNA sequencing (DNA-seq) with an average of 222 million 150-base pair (bp) paired-end reads (average insert size of 500 bp, resulting in an average of 76-fold coverage) and 77.8 million 150-bp mate-pair reads (average insert size of 2 kb, averaging 22-fold coverage) per individual. We also generated, on average, 26.6 million 75-bp paired-end RNA-seq reads for each individual.

Previous work on the sex chromosomes of these species showed evidence for male heterogametic systems in *P. wingei* (48), *P. picta* (50), and *G. holbrooki* (51), and a female heterogametic system in *P. latipinna* (52, 53). For each target species, we built a scaffold-level de novo genome assembly using SOAPdenovo2 (54) (*SI Appendix*, Table S2). Each assembly was constructed using the reads from the homogametic sex only in order to prevent coassembly of X and Y reads. This allowed us to later assess patterns of sex chromosome divergence based on differences between the sexes in read mapping efficiency to the genome (detailed below).

To obtain scaffold positional information for each species, we used the reference-assisted chromosome assembly (RACA) algorithm (55), which integrates comparative genomic data, through pairwise alignments between the genomes of a target, an outgroup (*Oryzias latipes* in this case), and a reference species (*Xiphophorus hellerii*), together with read mapping information from both sexes, to order target scaffolds into predicted chromosome fragments (*Materials and Methods* and *SI Appendix*, Table S2). RACA does not rely solely on sequence homology to the *X. hellerii* reference genome as a proxy for reconstructing the chromosomes in the target species, and instead incorporates read mapping and outgroup information from *O. latipes* (56) as well. This minimizes mapping biases that might result from different degrees of phylogenetic similarity of our target species to the reference, *X. hellerii*. Using RACA, we reconstructed chromosomal fragments in each target genome and identified syntenic blocks (regions that maintain sequence similarity and order) across the chromosomes of the target and reference species. This provided a comparison at the sequence level for each target species with reference genome and positional information of scaffolds in chromosome fragments.

### Extreme Heterogeneity in Sex Chromosome Differentiation Patterns.

For each target species, we used differences between males and females in genomic coverage and single-nucleotide polymorphisms (SNPs) to identify nonrecombining regions and strata of divergence. Additionally, we used published coverage and SNP density data in *P. reticulata* for comparative analyses (47).

In male heterogametic systems, nonrecombining Y degenerate regions are expected to show a significantly reduced coverage in males compared with females, as males have only 1 X chromosome, compared with 2 in females. In contrast, autosomal and undifferentiated sex-linked regions have an equal coverage between the sexes. Thus, we defined older nonrecombining strata of divergence as regions with a significantly reduced male-to-female coverage ratio compared with the autosomes.

Additionally, we used SNP densities in males and females to identify younger strata, representing earlier stages of sex chromosome divergence. In XY systems, regions that have stopped recombining more recently but that still retain high sequence similarity between the X and the Y show an increase in male SNP density compared with females, as Y reads, carrying Y-specific polymorphisms, still map to the homologous X regions. In contrast, we expect the opposite pattern of lower SNP density in males relative to females in regions of substantial Y degeneration, as the X in males is effectively hemizygous (the Y copy is lost or exhibits substantial sequence divergence from the X orthology).

**Fig. 1.**
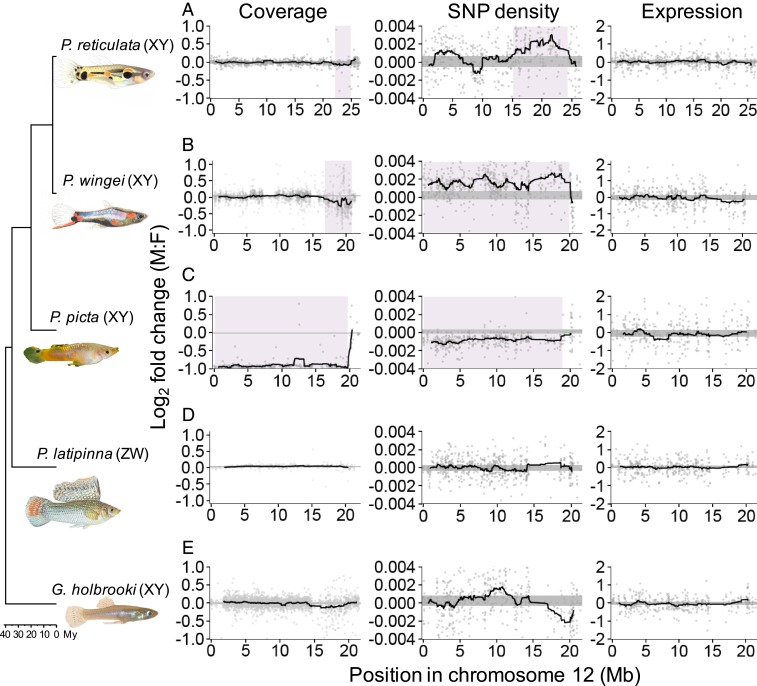
Differences between the sexes in coverage, SNP density, and expression across the guppy sex chromosome (*P. reticulata* chromosome 12) and syntenic regions in each of the target species. *X. hellerii* chromosome 8 is syntenic, and inverted, to the guppy sex chromosome. We used *X. hellerii* as the reference genome for our target chromosomal reconstructions. For consistency and direct comparison to *P. reticulata,* we used the *P. reticulata* numbering and chromosome orientation. Moving average plots show male-to-female differences in sliding windows across the chromosome in *P. reticulata* (*A*), *P. wingei* (*B*), *P. picta* (*C*), *P. latipinna* (*D*), and *G. holbrooki* (*E*). The 95% confidence intervals based on bootsrapping autosomal estimates are shown by the horizontal gray-shaded areas. Highlighted in purple are the nonrecombining regions of the *P. reticulata*, *P. wingei*, and *P. picta* sex chromosomes, identified through a significant deviation from the 95% confidence intervals.

Previous studies have suggested a very recent origin of the *P. reticulata* sex chromosome system based on its large degree of homomorphism and the limited expansion of the Y-specific region (47, 48). Contrary to these expectations, our combined coverage and SNP density analysis indicates that *P. reticulata*, *P. wingei*, and *P. picta* share the same sex chromosome system (Fig. 1 and *SI Appendix*, Figs. S1 and S2), revealing an ancestral system that dates back to at least 20 mya (57). Our findings suggest a far higher degree of sex chromosome conservation in this genus than we expected, based on the small nonrecombining region in *P. reticulata* in particular (47) and the high rate of sex chromosome turnover in fish in general (58, 59). By contrast, in the *Xiphophorous* and *Oryzias* genera, sex chromosomes have evolved independently between sister species (26, 60), and there are even multiple sex chromosomes within *Xiphophorous maculatus* (61).

In addition to the unexpected conservation of this poeciliid sex chromosome system, we observe extreme heterogeneity in patterns of X/Y differentiation across the 3 species. The *P. wingei* sex chromosomes have a similar, yet more accentuated, pattern of divergence compared with *P. reticulata* (Fig. 1 *A* and *B*). The nonrecombining region appears to span the entire *P. wingei* sex chromosomes, and, similar to *P. reticulata*, we can distinguish 2 evolutionary strata: an older stratum (17 to 20 megabases [Mb]), showing significantly reduced male coverage, and a younger nonrecombining stratum (0 to 17 Mb), as indicated by elevated male SNP density without a decrease in coverage (Fig. 1*B*). The old stratum has possibly evolved ancestrally to *P. wingei* and *P. reticulata*, as its size and estimated level of divergence appear to be conserved in the 2 species. The younger stratum, however, has expanded substantially in *P. wingei* relative to *P. reticulata* (47). These findings are consistent with the expansion of the heterochromatic block (48) and the large-scale accumulation of repetitive elements on the *P. wingei* Y chromosome (49).

More surprisingly, however, is the pattern of sex chromosome divergence that we recover in *P. picta*, which shows an almost 2-fold reduction in male-to-female coverage across the entire length of the sex chromosomes relative to the rest of the genome (Fig. 1*C*). This indicates not only that the Y chromosome in this species is completely nonrecombining with the X but also that the Y chromosome has undergone significant degeneration. Consistent with the notion that genetic decay on the Y chromosome will produce regions that are effectively hemizygous, we also recover a significant reduction in male SNP density (Fig. 1*C*). A limited pseudoautosomal region still remains at the far end of the chromosome, as both the coverage and SNP density patterns in all 3 species suggest that recombination persists in that area. As transitions from heteromorphic to homomorphic sex chromosomes are not uncommon in fish and amphibians (59), it is also possible, though less parsimonious, that the ancestral sex chromosome resembles more the structure found in *P. picta* and that the sex chromosomes in *P. wingei* and *P. reticulata* have undergone a transition to homomorphism.

**Fig. 2.**
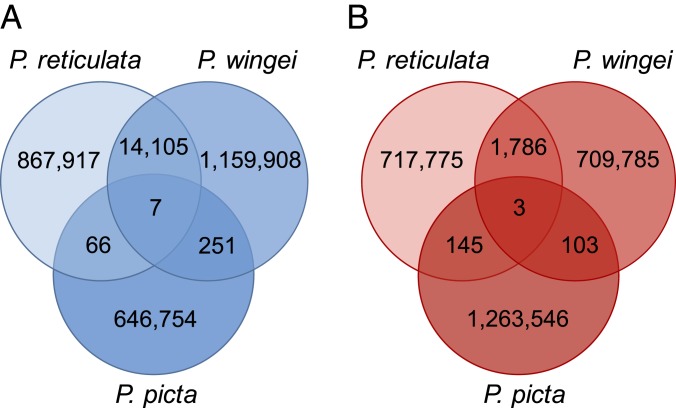
Number of shared *k*-mers across *P. reticulata*, *P. wingei*, and *P. picta*. Species-specific and shared male-unique *k*-mer (Y-mer) counts (*A*) and female-unique *k*-mer counts (*B*) are shown.

In order to identify the ancestral Y region, we used *k*-mer analysis across *P. reticulata, P. wingei*, and *P. picta*, which detects shared male-specific *k*-mers, often referred to as Y-mers. Using this method, we have previously identified shared male-specific sequences between *P. reticulata* and *P. wingei* (49) (Fig. 2). Curiously, we recovered here very few shared Y-mers across all 3 species (Fig. 2), which suggests 2 possible scenarios in the evolution of *P. picta* sex chromosomes. It is possible that sex chromosome divergence began independently in *P. picta* compared with *P. reticulata* and *P. wingei*. Alternatively, the ancestral Y chromosome in *P. picta* may have been largely lost via deletion, resulting in either a very small Y chromosome or an X0 system. To test for these alternative hypotheses, we reran the *k*-mer analysis in *P. picta* alone. We recovered almost twice as many female-specific *k*-mers than Y-mers in *P. picta* (Fig. 2), which indicates that much of the Y chromosome is indeed missing. This is consistent with the coverage analysis (Fig. 1*C*), which shows that male coverage of the X is half that of females, consistent with large-scale loss of homologous Y sequence.

We also used differences in coverage and SNP density between males and females to identify the sex chromosomes in the more distantly related *P. latipinna* and *G. holbrooki* (*SI Appendix*, Figs. S3 and S4). The coverage could suggest that the same region is the sex chromosome in both *P. reticulata* and *G. holbrooki*, as we find a small decrease in *G. holbrooki* male coverage toward the end of the chromosome (Fig. 1*E*). However, the SNP density patterns in *G. holbrooki* are not consistent with this finding, and therefore we cannot definitely conclude whether this is indeed the nonrecombining region. Importantly, the sex chromosome appears to have evolved independently in *P. latipinna*, as we cannot identify any areas of divergence or restricted recombination on the homolog of the *P. reticulata* sex chromosome in this species (Fig. 1*D*). Lack of conservation of the sex chromosome system is not unexpected for *P. latipinna*, as this species has evolved a female heterogametic system. Regardless, the sex chromosomes in *P. latipinna* and *G. holbrooki* are largely undifferentiated, and further linkage mapping of phenotypic sex is required to definitely determine the nonrecombining region.

### Y Degeneration and Dosage Compensation in *P. picta*.

**Fig. 3.**
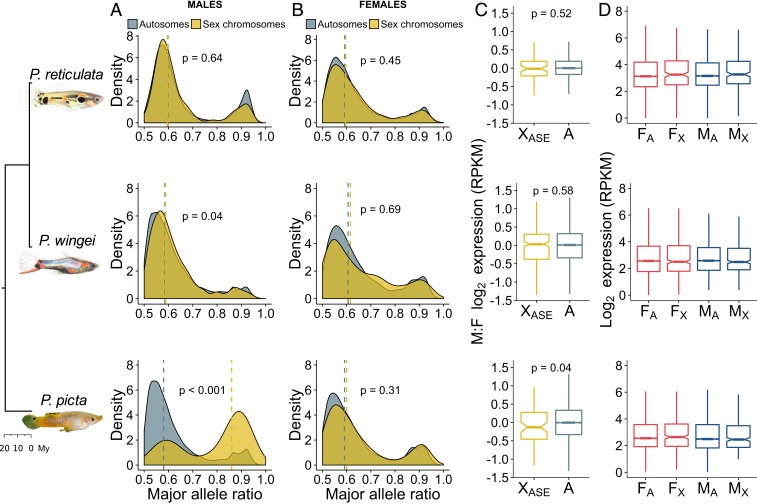
Patterns of gene expression and ASE. Density plots show the distribution of the major allele frequency of autosomal (gray) and sex chromosome (yellow) genes in males (*A*) and females (*B*) of each species. Vertical dotted lines indicate median values, and *P* values are based on Wilcoxon rank sum tests. (*C*) Boxplots show differences in log_2_ expression between the sexes (male/female) for autosomal genes (gray) and sex chromosome genes with an ASE pattern in males (yellow). *P* values are based on Wilcoxon rank sum tests. (*D*) Boxplots show average male (blue) and female (red) log_2_ expression for autosomes (A) and the nonrecombining region of the sex chromosomes (X) in each species. F, female; M, male.

To investigate the extent of Y gene activity decay in our target species, we estimated allele-specific expression (ASE) patterns from RNA-seq data (Fig. 3 *A* and *B*). If the X and Y chromosomes are transcriptionally active at the same level, we expect a probability of around 0.5 of recovering reads from either chromosome. Conversely, regions of Y gene activity decay should be reflected by a significantly unbalanced contribution from the 2 sex chromosomes to the overall expression of heterozygous sites in males.

In *P. picta*, we found that males and females have a similar proportion of autosomal genes with heterozygous sites (56% in females and 68% in males). However, of the 363 sex-linked genes in *P. picta*, males have only 96 (27%) genes with Y-linked SNPs, while females have 177 (49%) genes with heterozygous sites. This likely indicates that many of the genes on the sex chromosomes in males are X-hemizygous. Of the 96 sex-linked genes with a transcriptionally active Y-linked copy, 77 show significant ASE in males (Fig. 3*A*). Indeed, our allelic differential expression analysis revealed that a significantly larger proportion of heterozygous sites show an ASE pattern on the sex chromosomes than on the autosomes in *P. picta* males [χ^2^(1) = 41.3710 and *P* < 0.001; Fig. 3*A*]. In contrast, in *P. picta* females and in both males and females of *P. reticulata* and *P. wingei*, the majority of genes throughout the genome show equal transcription between the maternal and paternal chromosomes (Fig. 3 *A* and *B*). These findings confirm extensive Y gene activity decay in *P. picta*.

Given the profound degeneration of the Y chromosome in *P. picta*, we would expect the overall gene expression for the sex chromosomes to be roughly halved in males compared with females. Remarkably, despite these expectations, we found no significant reduction in average gene expression on the sex chromosomes in males when compared with either the sex chromosomes in females (*P* = 0.09, Wilcoxon signed rank test) or the autosomes in males (*P* = 0.94, Wilcoxon rank sum test; Fig. 3*D*). This finding confirms the presence of a chromosome-wide dosage compensation mechanism in *P. picta*, acting to counteract the imbalance in gene dose in males. Moreover, sex-linked genes with an ASE pattern in males show only a marginally significant decrease in male-to-female expression compared with autosomal genes (*P* = 0.04, Wilcoxon rank sum test; Fig. 3*C*). This likely represents a functional dosage compensation mechanism that is far more effective than the partial, localized dosage compensation recorded in other fish species (35–38).

We additionally tested for an enrichment of gene ontology (GO) terms for genes in the nonrecombining region of the sex chromosomes compared with genes expressed in the remainder of the genome; however, we found no GO terms with significant (*P* < 0.001) enrichment in any of the species. Examining differential expression patterns across each target genome revealed no evidence of sex chromosome sexualization, as levels of sex-biased gene expression are not significantly different between the autosomes and the sex chromosomes (*SI Appendix*, Table S3). However, we noticed a slight deficit in male-biased genes on the *P. picta* sex chromosomes relative to the autosomes. This is possibly a consequence of Y degeneration and dosage compensation in this system, as it is more difficult for genes on the sex chromosomes to achieve male-biased expression compared with genes on the autosomes, given that they are already hyperexpressed as a result of dosage compensation (62).

## Concluding Remarks

Our comparative analyses reveal a striking heterogeneity in the degree of recombination suppression and Y chromosome degeneration across poeciliid species with a shared sex chromosome system. Through multiple independent lines of evidence, including sequence coverage, *k*-mer analysis, and ASE patterns, we show a profound degeneration of the Y chromosome in *P. picta*. Remarkably, the hemizygosity of the X in males has led to the evolution of a functional, chromosome-wide dosage compensation in this species, a mechanism not previously reported in fish. Our findings highlight the importance of comparative studies of sex chromosome differentiation within clades and suggest that fish may harbor extensive variation in sex chromosome evolution.

## Materials and Methods

We used DNA-seq and RNA-seq data from males and females from 4 guppy species (*P. wingei* from our laboratory population, *P. picta* from Guyana, and *P. latipinna* and *G. holbrooki* from Florida). All samples were collected in accordance with ethical guidelines under Florida permit (FNW17-10), St. Mark’s Refuge permit (FF04RFSM00-17-09), and Environmental Protection Agency of Guyana permit (Permit 120616 SP: 015). For each species, we constructed scaffold-level de novo genome assemblies using SOAPdenovo2 (54). To improve assembly contiguity and reconstruct chromosome fragments for each species, we followed the University of California, Santa Cruz chains and nets pipeline (63), together with the RACA algorithm (55). We first obtained pairwise alignments between our target genomes, a reference genome (*X. hellerii*), and an outgroup genome (*O. latipes*) using LASTZ v1.04 (64) and the chains and nets pipeline (*SI Appendix*). We also obtained paired-end and mate-pair target read mappings to the de novo assemblies using Bowtie2 (65). RACA then incorporated information from both the pairwise alignments and the read mapping data to merge target scaffolds into longer predicted chromosome fragments for each target species. We were thus able to assign chromosomal positional information to scaffolds and assess synteny of sex chromosome systems across the target species.

We next estimated the extent of sex chromosome differentiation in each species. We mapped DNA-seq and RNA-seq reads to the de novo scaffolds with chromosomal annotation from RACA, and obtained sequence coverage, polymorphism, and expression data. To assess patterns of sex chromosome divergence and recombination suppression, we compared coverage and SNP density differences between males and females within each species. We then used *k*-mer analysis to identify ancestral Y sequence (*SI Appendix*). Additionally, we estimated the extent of genetic decay on the sex-limited chromosome of each system and tested for the presence of dosage compensation using expression differences between the sexes and ASE patterns from RNA-seq data. Full methods are included in *SI Appendix*.

## Supplementary Material

Supplementary File
