# Supplementary material for: Extreme heterogeneity in sex chromosome differentiation and dosage compensation in livebearers
Source: Proc Natl Acad Sci U S A. 2019 Sep 4;116(38):19031–6. doi: 10.1073/pnas.1905298116 (PMC6754558; doi:10.1073/pnas.1905298116)
Supplement: Supplementary File [file pnas.1905298116.sapp.pdf]

PNAS
www.pnas.org

Supplementary Information for

Extreme heterogeneity in sex chromosome differentiation and dosage compensation in livebearers

Iulia Darolti[a,1], Alison E. Wright[b], Ben A. Sandkam[c], Jake Morris[a], Natasha I. Bloch[d], Marta Farré[e], Rebecca C. Fuller[f], Godfrey R. Bourne[g], Denis M. Larkin[h], Felix Breden[i], Judith E. Mank[a,c,j]

[a]Department of Genetics, Evolution and Environment, University College London, UK; [b]Department of Animal and Plant Sciences, University of Sheffield, UK; [c]Department of Zoology, University of British Columbia, Canada; [d]Department of Biomedical Engineering, University of Los Andes, Colombia; [e]School of Biosciences, University of Kent, UK; [f]Department of Animal Biology, University of Illinois at Urbana-Champaign, USA; [g]Department of Biology, University of Missouri St. Louis, USA; [h]Royal Veterinary College, UK; [i]Department of Biological Science, Simon Fraser University, Canada; [j]Department of Organismal Biology, Uppsala University, Sweden

*Correspondence to: iulia.darolti.15@ucl.ac.uk

This PDF file includes:

Supplementary text
Figs. S1 to S4
Tables S1 to S3
References for SI reference citations

www.pnas.org/cgi/doi/10.1073/pnas.1905298116

Supplementary Materials and Methods

Sample collection and sequencing. We collected adult male and female individuals from four guppy species (*Poecilia wingei* from our laboratory population, *Poecilia picta* from Guyana, *Poecilia latipinna* and *Gambusia holbrooki* from Florida, USA). We chose these samples in order to obtain an even phylogenetic distribution. The species we assessed exhibit clear somatic dimorphisms, including coloration and size, in addition to gonadal differences. Most notably, females possess an enlarged abdomen and anal fin. In males, the anal fin is modified to form a gonopodium (i.e. and intromittent organ), which is clearly visible. Phenotypic sex was determined at the time of collection based on these measures. There were no intermediate or ambiguous individuals collected and sex was clearly visible and concordant across both somatic and phenotypic traits in all samples. All samples were collected in accordance within ethical guidelines. *P. latipinna* and *G. holbrooki* were collected under Florida permit FNW17-10 and St. Mark's Refuge permit FF04RFSM00-17-09. *P. picta* was collected under permit from the Environmental Protection Agency of Guyana (Permit 120616 SP: 015). *P. wingei* was collected from our lab population, a colony of a strain maintained by a UK fish fancier.

From each species, we immediately stored head and tail samples from three males and three females in ethanol and, RNAlater, respectively. We extracted DNA from heads with the DNeasy Blood and Tissue Kit (Qiagen) and RNA from tails with the RNeasy Kit (Qiagen), following the manufacturer's instructions. Library preparation and sequencing were performed at The Wellcome Trust Centre for Human Genetics, University of Oxford, following standard protocols and using the Illumina HiSeq 4000 platform. Genomic DNA was used to construct paired-end (PE) sequencing libraries with short insert sizes (average insert size 500bp) and mate-pair (MP) libraries with long insert sizes (average insert size 2kb) for each individual. The Nextera Mate Pair Sample Preparation Kit was used for preparing mate-pair libraries. We assessed data quality with FastQC v0.11.3 (www.bioinformatics.babraham.ac.uk/projects/fastqc/) and used Trimmomatic v0.36 (1) to trim reads. For both DNA-seq and RNA-seq reads we removed adaptor sequences, regions of low Phred score (reads with average Phred score <15 in sliding

windows of four bases and reads with leading/trailing bases with a Phred score < 3) and short reads (if either read in a pair was shorter than 50bp).

Genome assembly. We first corrected the reads using Quake v0.3.5 (2) and estimated the optimal assembly *k*-mer length using KmerGenie v1.6741 (3). We then used SOAPdenovo v2.04 (4) to construct female *de novo* genome assemblies for *P. wingei*, *P. picta* and *G. holbrooki* and a male assembly for *P. latipinna*, using both the paired-end and mate-pair reads (Table S2). The paired-end reads were used for both the contig and scaffolding steps of the assembly process, while the mate-pair reads were only used for scaffolding. Additionally, we used the SOAPdenovo GapCloser module to close the gaps resulting from the assembly scaffolding step. Finally, we removed sequences shorter than 1kb from the assemblies.

To improve assembly contiguity and to reconstruct chromosomal fragments for each species, we followed the UCSC chains and nets pipeline from the kentUtils software suite (5) before employing the Reference-Assisted Chromosome Assembly (RACA) algorithm (6). The chains and nets pipeline is designed for building pairwise nucleotide alignments and bridging gaps between pairwise syntenic blocks to construct larger structures (5). A chain alignment represents an ordered pairwise sequence alignment between two species. A net alignment represents a collection of chains within a genome region, ordered in a hierarchical manner based on synteny scoring. The RACA algorithm incorporates the pairwise alignment files, together with read mapping information to identify syntenic fragments (regions which maintain sequence similarity and order) across the species used. RACA then estimates adjacency between syntenic fragments in each target genome to reconstruct predicted chromosome fragments (PCFs) for each target species (6).
First, for each species, we carried out DNA-seq read mappings to the *de novo* assemblies using Bowtie2 v2.3.3.1 (7), reporting concordant mappings only (--no-discordant option) and using the appropriate mate orientations according to the insert size of the libraries (--fr option for short-insert libraries and --rf option for long-insert libraries). The resulting alignments were converted into the RACA-specific input format (script available on the RACA website http://bioen-compbio.bioen.illinois.edu/RACA/).

We also obtained pairwise alignments using LASTZ (www.bx.psu.edu/%7Ersharris/lastz/; parameters C=0, E=30, H=2000, K=3000, L=3000, O=400, M=50) between a reference species (here we used the *X. hellerii* genome, obtained from NCBI GenBank Xiphophorus_hellerii-4.0, assembly accession GCA_003331165.1), the target species and an outgroup species (here we used the Medaka, *Oryzias latipes*, genome, obtained from GenBank ASM223467v1, assembly accession GCA_002234675.1). We then converted these alignments into chains and nets formats following the UCSC axtChain (-minScore=1000, -linearGap=medium), chainAntiRepeat, chainSort, chainPreNet and netSyntenic tools (5). The syntenic chains and nets fragments, together with the paired-end alignments, were used as input files for RACA (resolution=10000 for *P. picta* and *P. latipinna* and resolution=1000 for *P. wingei* and *G. holbrooki*). For each target species, RACA ordered and oriented target scaffolds into PCFs (Table S2), and we used this positional information of scaffolds in the genome for all further analyses.

Analysis of genomic coverage. For each species, using BWA v0.7.12 (8), we mapped male and female paired-end DNA-seq reads to the *de novo* scaffolds with positional annotation from RACA, following the aln and sampe alignment steps, and extracted uniquely mapping reads. We then used soap.coverage v2.7.9 (http://soap.genomics.org.cn/) to calculate the coverage (number of times each site was sequenced divided by the total number of sequenced sites) of each scaffold in each sample. For each scaffold, we calculated the male to female (M:F) fold change coverage as $\log_2$(average male coverage) – $\log_2$(average female coverage).

SNP density analysis. For each species, using Bowtie1 v1.1.2 (7), we mapped male and female paired-end DNA-seq reads to the *de novo* scaffolds with positional annotation from RACA, generating map format output files. We sorted the map files by scaffold and converted them into profiles, which represent counts for each of the four nucleotide bases, for each individual using bow2pro v0.1 (http://guanine.evolbio.mpg.de/). For each site, we applied a minimum coverage threshold of 10 and called SNPs as sites with a major allele frequency of 0.3x the total site coverage. We obtained gene information

through the expression analysis detailed below and for each gene we calculated the average SNP density as the number of SNPs divided by the number of filtered sites. We excluded SNPs outside of genic regions. For each gene we then calculated M:F fold change SNP density as $\log_2$(average male SNP density) – $\log_2$(average female SNP density).

Detection of sex chromosome non-recombining regions and strata of divergence. We used the fold change coverage and SNP density estimates to distinguish regions that are homologous and recombining between the sex chromosomes from regions that show full or even partial sex chromosome divergence, and which are hence non-recombining. For each species, we generated 95% confidence intervals based on bootstrapping autosomal M:F coverage ratios and autosomal M:F SNP density ratios separately. For XY systems, we defined non-recombining, older strata of divergence as regions with a significant decrease in M:F coverage ratio outside the 95% confidence interval. In addition, we defined younger strata of divergence as regions with no reduction in male coverage but with a significant increase in M:F SNP density ratio outside the 95% confidence interval. Conversely, for ZW systems, a significant increase in M:F coverage ratio and a significant decrease in M:F SNP density ratio are expected for older and, respectively, younger regions of divergence.

Gene expression analysis. For each species, using HISAT2 v2.0.4 (9), we mapped male and female RNA-seq reads to scaffolds with positional annotation from RACA, reporting paired (--no-mixed) and concordant (--no-discordant) mappings only and tailoring the alignments for downstream transcript assembly (--dta). We used SAMtools to sort by coordinate and bam convert the sam output files. For each sample, we then used StringTie (10) to obtain transcripts in a GTF file format, which we then merged to assemble a non-redundant set of transcripts for each species. Before further analyses, we filtered the merged GTF file for non-coding RNA (ncRNA) by using BEDtools getfasta (11), extracted target transcript sequences and removed transcripts with BLAST hit to ncRNA sequences from *Poecilia formosa* (PoeFor_5.1.2), *Oryzias latipes* (MEDAKA1),

Gasterosteus aculeatus (BROADS1), and *Danio rerio* (GRCz10), obtained from Ensembl 84 (12).

For each species, we estimated gene expression by extracting read counts for each gene using HTSeq-count (13) and the ncRNA filtered transcriptome. We only kept genes that were placed on scaffolds with positional information on PCFs. For these genes, we converted read counts to RPKM values with edgeR (14), normalised with TMM, and applied a minimum expression threshold of 2RPKM in half or more of the individuals in one sex. For each gene we then calculated M:F fold change expression as $\log_2$(average male expression) – $\log_2$(average female expression).

We identified sex-biased genes in EdgeR using a minimum of two-fold differential expression ($\log_2$ M:F RPKM > 1 for male-biased genes and < -1 for female-biased genes) and a significant p value ($p_{adj} < 0.05$ based on FDR correction for multiple testing (15)).

We tested for an enrichment of GO terms in the non-recombining regions of the sex chromosomes relative to the rest of the genome in each species. We first extracted the longest isoform for each gene from the *Danio rerio* (GRCz10) coding sequences from Ensembl 84. We then BLASted longest isoforms from each of our target gene sets to the *D. rerio* sequences with BLASTn v2.3.0 (16), using an e-value cutoff of $10e^{-10}$ and a minimum percentage identity of 30%. For genes with multiple alignment hits, we chose the top blast hit based on the highest BLAST score. We then compared *D. rerio* orthologues for genes in the non-recombining regions with those for genes in the rest of the genome using GOrilla (17).

***k*-mer analysis.** In order to identify shared Y sequence across *P. reticulata*, *P. wingei* and *P. picta*, we followed a *k*-mer analysis method previously described in Morris et al. 2018 (18). We have previously used this approach to successfully identify shared Y sequence between *P. reticulata* and *P. wingei* (18). Briefly, here we used the HAWK pipeline (19) to count *k*-mers from paired-end DNA-seq reads and identify unique *k*-mers for each sex

in each species. Across all the species, we then identified shared female unique *k*-mers and shared male unique *k*-mers, referred to as Y-mers (18).

Allele-specific expression (ASE) analysis. In order to estimate ASE patterns from RNA-seq data, we tailored previously published pipelines (20, 21). For each species, we called SNPs separately for males and females using SAMtools mpileup and varscan (22), with parameters --min-coverage 2, --min-ave-qual 20, --min-freq-for-hom 0.90, and excluding triallelic SNPs and Ns. Additionally, we removed SNPs that were not located within genic regions from the final filtered gene dataset. To exclude potential sequencing errors from our SNP dataset, we applied coverage filtering thresholds (20, 21). Firstly, we set a minimum site coverage of 15 reads (the sum of major and minor alleles), as a power analysis indicated that at a minimum coverage of 15 reads we have a 78% power to detect a signal of allele-specific expression. Secondly, we applied a variable coverage filter that accounts for the change in the likelihood of sequencing errors at different coverage levels (accounting for an error rate of 1 in 100 and a maximum coverage for a given site of 100,000 (20)). Lastly, to avoid the potential bias in our ASE estimations from the preferential assignment of reads to the reference allele (23), we removed clusters of more than 5 SNPs in 100 bp windows.

If genes have biallelic expression, meaning that alleles from both chromosomes are expressed at the same level, we expect a probability of around 0.5 of recovering reads from either chromosome. For each SNP in the final filtered dataset we tested for ASE by identifying significant deviations from the expected probability of 0.5 using a two-tailed binomial test ($p < 0.05$). We corrected for multiple testing when running binomial tests on autosomal SNPs. Additionally, we called SNPs as ASE if a minimum of 70% of the reads stemmed from one of the chromosomes. We called genes as ASE if they had at least one SNP with a consistent ASE pattern across all heterozygous samples. We tested for significant differences in ASE patterns between the sexes and between the autosomes and the sex chromosomes using chi-square tests.

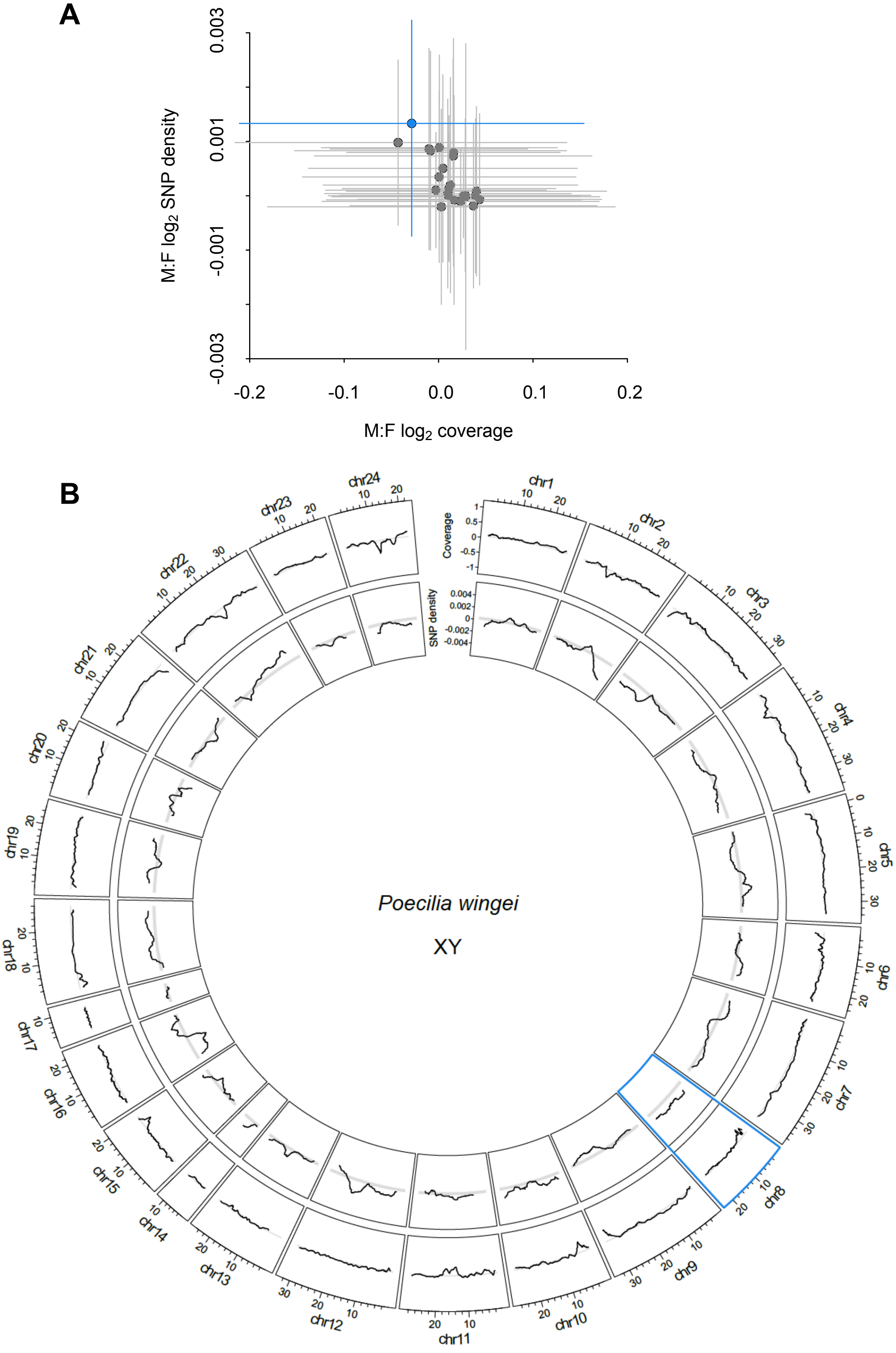
A
0.003
0.001
-0.001
-0.003
M:F log2 SNP density
-0.2
-0.1
0.0
0.1
0.2
M:F log2 coverage
B
Poecilia wingei
XY
Coverage
SNP density
chr1
chr2
chr3
chr4
chr5
chr6
chr7
chr8
chr9
chr10
chr11
chr12
chr13
chr14
chr15
chr16
chr17
chr18
chr19
chr20
chr21
chr22
chr23
chr24

Fig. S1. Coverage and SNP density differences between the sexes (male:female) for *P. wingei* scaffolds placed by RACA on the reference *X. hellerii* chromosomes. (A) Average coverage and SNP density fold change for each chromosome. Shown in blue is *X. hellerii* chromosome 8, which is syntenic to the guppy sex chromosome (*P. reticulata* chromosome 12), and constitutes the sex chromosome in *P. wingei.* Interquartile ranges are represented by the vertical and horizontal lines. (B) Circos plot showing moving average of $\log_2$ M:F coverage (outer ring) and $\log_2$ M:F SNP density (inner ring) fold change across each chromosome. Highlighted in blue is the XY sex chromosome in *P. wingei*. Horizontal grey-shaded areas represent the 95% confidence intervals based on bootstrap estimates across the genome, excluding the sex chromosome.

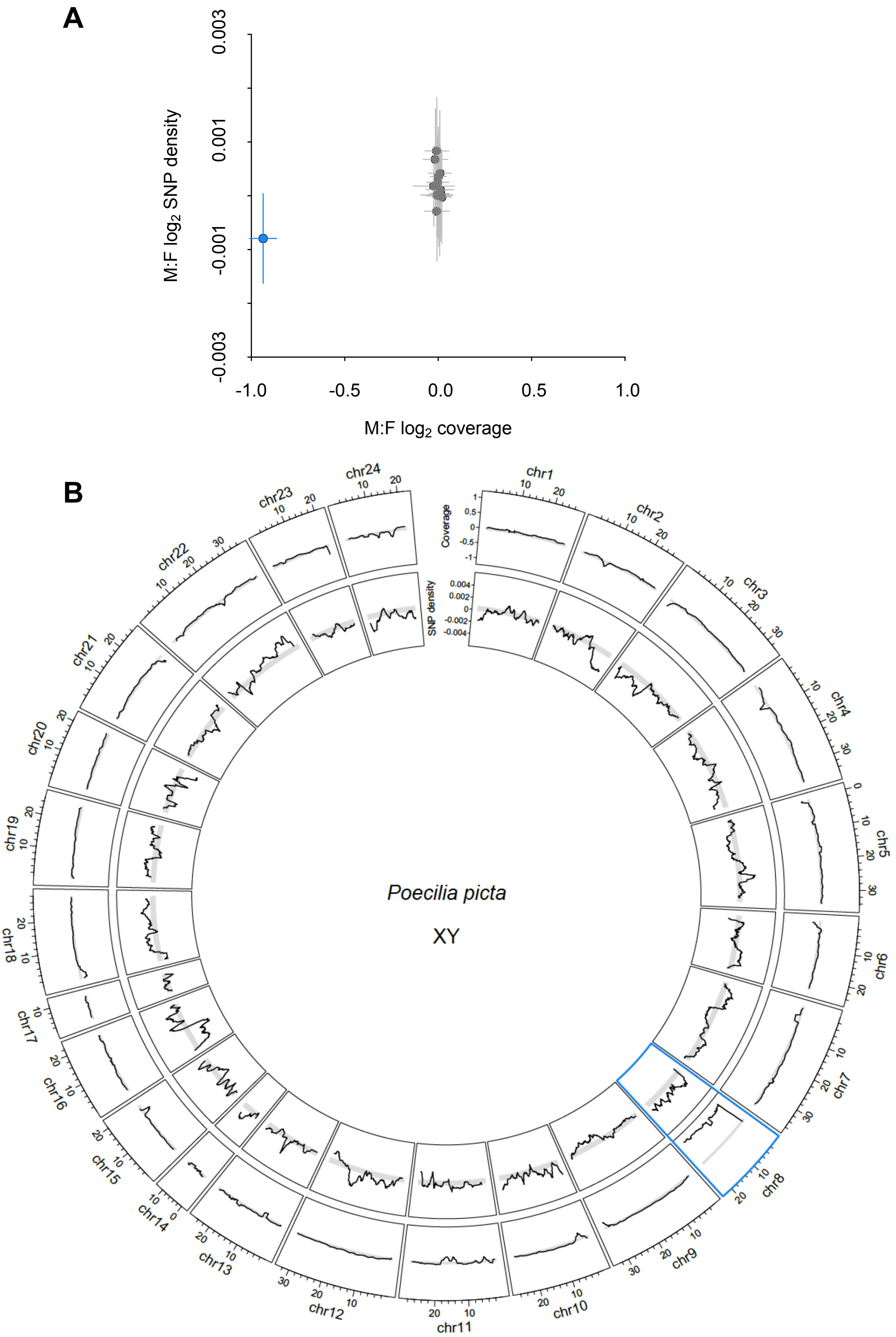
A
M:F log2 SNP density
M:F log2 coverage
0.003
0.001
-0.001
-0.003
-1.0
-0.5
0.0
0.5
1.0
B
Coverage
SNP density
Poecilia picta
XY
chr1
chr2
chr3
chr4
chr5
chr6
chr7
chr8
chr9
chr10
chr11
chr12
chr13
chr14
chr15
chr16
chr17
chr18
chr19
chr20
chr21
chr22
chr23
chr24

Fig. S2. Coverage and SNP density differences between the sexes (male:female) for *P. picta* scaffolds placed by RACA on the reference *X. hellerii* chromosomes. (A) Average coverage and SNP density fold change for each chromosome. Shown in blue is *X. hellerii* chromosome 8, which is syntenic to the guppy sex chromosome (*P. reticulata* chromosome 12), and constitutes the sex chromosome in *P. picta.* Interquartile ranges are represented by the vertical and horizontal lines. (B) Circos plot showing moving average of $\log_2$ M:F coverage (outer ring) and $\log_2$ M:F SNP density (inner ring) fold change across each chromosome. Highlighted in blue is the XY sex chromosome in *P. picta*. Horizontal grey-shaded areas represent the 95% confidence intervals based on bootstrap estimates across the genome, excluding the sex chromosome.

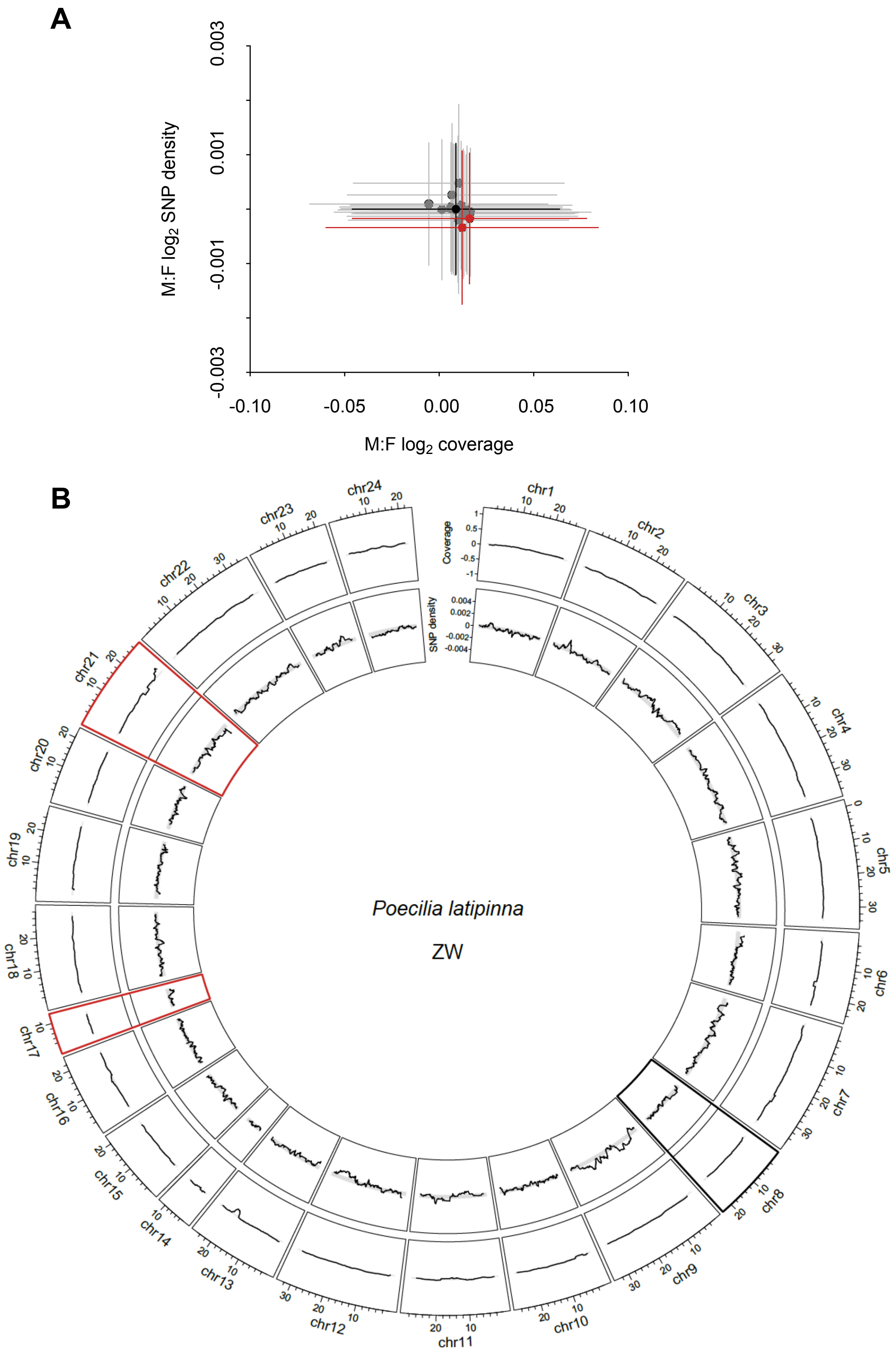
A
M:F log2 SNP density
M:F log2 coverage
0.003
0.001
-0.001
-0.003
-0.10
-0.05
0.00
0.05
0.10
B
Poecilia latipinna
ZW
Coverage
SNP density
chr1
chr2
chr3
chr4
chr5
chr6
chr7
chr8
chr9
chr10
chr11
chr12
chr13
chr14
chr15
chr16
chr17
chr18
chr19
chr20
chr21
chr22
chr23
chr24

Fig. S3. Coverage and SNP density differences between the sexes (male:female) for *P. latipinna* scaffolds placed by RACA on the reference *X. hellerii* chromosomes. (A) Average coverage and SNP density fold change for each chromosome. Shown in red are chromosomes 17 and 21, ZW sex chromosome candidates for *P. latipinna*. Chromosome 8, which is syntenic to the guppy sex chromosome (*P. reticulata* chromosome 12), is shown in black. Interquartile ranges are represented by the vertical and horizontal lines. (B) Circos plot showing moving average of $\log_2$ M:F coverage (outer ring) and $\log_2$ M:F SNP density (inner ring) fold change across each chromosome. Highlighted in red are the *P. latipinna* ZW sex chromosome candidates, as identified in (A). Horizontal grey-shaded areas represent the 95% confidence intervals based on bootstrap estimates across the genome, excluding the sex chromosome candidates.

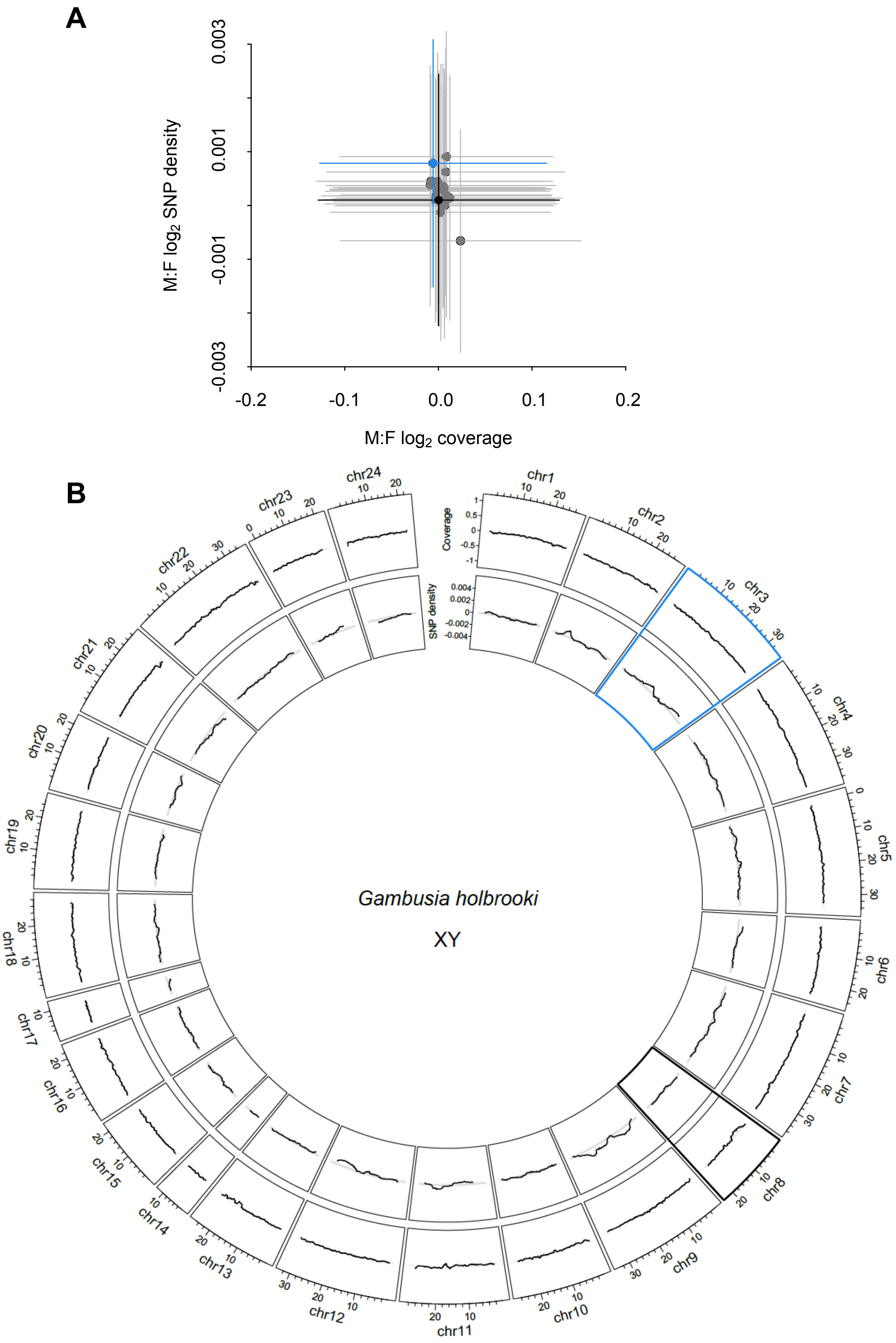

A
M:F log2 SNP density
M:F log2 coverage
0.003
0.001
-0.001
-0.003
-0.2
-0.1
0.0
0.1
0.2
B
Gambusia holbrooki
XY
Coverage
SNP density
chr1
chr2
chr3
chr4
chr5
chr6
chr7
chr8
chr9
chr10
chr11
chr12
chr13
chr14
chr15
chr16
chr17
chr18
chr19
chr20
chr21
chr22
chr23
chr24

Fig. S4. Coverage and SNP density differences between the sexes (male:female) for *G. holbrooki* scaffolds placed by RACA on the reference *X. hellerii* chromosomes. (A) Average coverage and SNP density fold change for each chromosome. Shown in blue is chromosome 3, an XY sex chromosome candidate for *G. holbrooki*. Chromosome 8, which is syntenic to the guppy sex chromosome (*P. reticulata* chromosome 12), is shown in black. Interquartile ranges are represented by the vertical and horizontal lines. (B) Circos plot showing moving average of $\log_2$ M:F coverage (outer ring) and $\log_2$ M:F SNP density (inner ring) fold change across each chromosome. Highlighted in blue is a *G. holbrooki* XY sex chromosome candidate, as identified in (A). Horizontal grey-shaded areas represent the 95% confidence intervals based on bootstrap estimates across the genome, excluding the sex chromosome candidate.

Table S1. Sequencing results for each sample.

Species (Treatment)	Sample no. (Sex)	Paired reads after trimming	% kept after trimming	Coverage
***Poecilia wingei* (DNA-seq PE)**	291 (F)	222,019,309	97.7	77X
	292 (F)	209,095,391	92.6	72X
	293 (F)	244,778,587	92.3	85X
	294 (M)	221,308,140	92.9	76X
	295 (M)	245,199,642	93.3	85X
	296 (M)	214,802,737	93.2	74X
***Poecilia picta* (DNA-seq PE)**	247 (F)	201,783,529	92.5	70X
	248 (F)	248,146,529	93.4	86X
	265 (F)	251,440,989	93.2	87X
	266 (M)	264,471,289	93.4	91X
	267 (M)	209,266,241	93.3	72X
	268 (M)	213,098,477	93.7	74X
***Poecilia latipinna* (DNA-seq PE)**	269 (F)	242,950,245	93.6	83X
	270 (F)	186,547,462	92.7	64X
	271 (F)	194,577,608	92.7	67X
	272 (M)	235,795,174	93.3	81X
	289 (M)	229,757,997	93.4	79X
	290 (M)	232,391,653	93.0	80X
***Gambusia holbrooki* (DNA-seq PE)**	241 (F)	217,994,173	93.8	75X
	242 (F)	193,263,881	93.5	67X
	243 (F)	229,309,343	93.3	79X
	244 (M)	195,792,613	93.4	68X
	245 (M)	194,586,542	93.6	67X
	246 (M)	220,591,540	93.4	76X
***Poecilia wingei* (DNA-seq MP)**	013 (F)	80,809,424	58.0	23X
	014 (F)	76,562,926	58.1	22X
	015 (F)	77,120,163	58.5	22X
	016 (M)	75,360,153	56.4	22X
	018 (M)	80,705,804	57.9	23X
	019 (M)	83,808,049	58.8	24X
***Poecilia picta* (DNA-seq MP)**	013 (F)	81,263,670	57.7	23X
	014 (F)	75,174,083	56.9	22X
	015 (F)	86,920,083	57.1	25X
	016 (M)	73,917,330	56.4	21X
	018 (M)	79,696,940	56.0	23X
	019 (M)	76,727,662	57.1	22X
	002 (F)	87,479,612	56.1	25X

***Poecilia latipinna* (DNA-seq MP)**	004 (F)	87,085,262	56.8	25X
	005 (F)	54,308,904	56.4	16X
	006 (M)	78,744,655	57.0	23X
	007 (M)	84,406,439	54.2	24X
	012 (M)	88,707,007	58.9	26X
***Gambusia holbrooki* (DNA-seq MP)**	002 (F)	82,118,221	66.3	23.6
	004 (F)	76,472,890	55.6	22.0
	005 (F)	77,475,370	54.2	22.3
	006 (M)	66,891,462	56.4	19.2
	007 (M)	72,014,055	56.5	20.7
	012 (M)	63,635,368	56.8	18.3
***Poecilia wingei* (RNA-seq)**	201 (F)	35,176,172	94.0	-
	202 (F)	47,040,049	94.4	-
	203 (F)	48,558,664	94.4	-
	265 (M)	44,255,632	94.2	-
	266 (M)	41,375,146	94.2	-
	267 (M)	42,277,857	93.9	-
***Poecilia picta* (RNA-seq)**	282 (F)	33,616,549	93.9	-
	284 (F)	43,438,223	94.3	-
	285 (M)	45,953,612	94.3	-
	286 (M)	39,836,450	94.0	-
	287 (M)	43,314,678	94.1	-
	302 (F)	48,435,135	94.0	-
***Poecilia latipinna* (RNA-seq)**	228 (F)	48,056,489	94.3	-
	229 (F)	34,836,324	94.3	-
	230 (M)	35,640,155	94.7	-
	231 (M)	34,564,529	93.8	-
	232 (M)	34,774,385	93.9	-
	288 (F)	50,234,040	94.0	-
***Gambusia holbrooki* (RNA-seq)**	204 (F)	38,909,731	94.5	-
	205 (F)	44,717,526	94.9	-
	206 (F)	45,915,199	97.7	-
	207 (M)	46,496,039	94.1	-
	208 (M)	42,781,352	94.3	-
	281 (M)	39,993,511	93.1	-

Table S2. Assembly statistics.

Species	Total assembly length (Mb)	N50 (kb)	No. *de novo* scaffolds	No. RACA Predicted Chromosome Fragments
P. wingei	795.5	14.6	120,169	400
P. picta	782.2	150.6	9,640	201
P. latipinna	787.5	90.3	13,851	255
G. holbrooki	617.8	6.1	137,790	27

Table S3. Differential gene expression results.

Species	Categories	Autosomes + PAR	Non-recombining region	Chi-square test
P. reticulata	Total genes	13,075	231	
	Sex-biased	531 (4.0%)	11 (4.8%)	$X^2(1) = 0.1183$, $p = 0.73$
	Male-biased	337 (2.6%)	7 (3.0%)	$X^2(1) = 0.0439$, $p = 0.83$
	Female-biased	194 (1.5%)	4 (1.7%)	$X^2(1) = 0.0009$, $p = 0.97$
P. wingei	Total genes	12,066	472	
	Sex-biased	775 (6.4%)	34 (7.2%)	$X^2(1) = 0.2889$, $p = 0.59$
	Male-biased	346 (2.9%)	16 (3.4%)	$X^2(1) = 0.2546$, $p = 0.61$
	Female-biased	429 (3.6%)	18 (3.8)	$X^2(1) = 0.0255$, $p = 0.87$
P. picta	Total genes	10,706	363	
	Sex-biased	2,176 (20.3%)	77 (21.2%)	$X^2(1) = 0.0729$, $p = 0.79$
	Male-biased	929 (8.7%)	29 (7.9.%)	$X^2(1) = 0.1070$, $p = 0.74$
	Female-biased	1,247 (11.6%)	48 (13.2%)	$X^2(1) = 0.5320$, $p = 0.47$

References

1. Lohse M, Bolger AM, Nagel A, Fernie AR, Lunn JE, Stitt M, Usadel B (2012) RobiNA: A user-friendly, integrated software solution for RNA-Seq-based transcriptomics. *Nucleic Acids Res.* 40:622-627.

2. Kelley DR, Schatz MC, Salzberg SL (2010) Quake: quality-aware detection and correction of sequencing errors. *Genome Biol* 11:R116.

3. Chikhi R, Medvedev P (2014) Informed and automated k-mer size selection for genome assembly. *Bioinformatics* 30(1):31-37.

4. Luo R, Liu B, Xie Y, Li Z, Huang W, Yuan J, He G, Chen Y, Pan Q, Liu Y, Tang J, Wu G, Zhang H, Shi Y, Liu Y, Yu C, Wang B, Lu Y, Han C, Cheung DW, Yiu SM, Peng S, Xiaoqian Z, Liu G, Liao X, Li Y, Yang H, Wang J, Lam TW, Wang J (2012) SOAPdenovo2: an empirically improved memory-efficient short-read de novo assembler. *Gigascience* 1(1):18.

5. Kent WJ, Baertsch R, Hinrichs A, Miller W, Haussler D (2003) Evolution's cauldron: duplication, deletion, and rearrangement in the mouse and human genomes. *Proc Natl Acad Sci USA* 100(20):11484-11489.

6. Kim J, Larkin DM, Cai Q, Asan, Zhang Y, Ge RL, Auvil L, Capitanu B, Zhang G, Lewin HA, Ma J (2013) Reference-assisted chromosome assembly. *PNAS* 110(5):1785-1790.

7. Langmead B, Trapnell C, Pop M, Salzberg SL (2009) Ultrafast and memory-efficient alignment of short DNA sequences to the human genome. *Genome Biol* 10(3):R25.

8. Li H, Durbin R (2009) Fast and accurate short read alignment with Burrows-Wheeler transform. *Bioinformatics* 25(14):1754-1760.

9. Kim D, Langmead B, Salzberg SL (2015) HISAT: A fast spliced aligner with low memory requirements. *Nature Methods* 12(4):357-360.

10. Pertea M, Pertea GM, Antonescu CM, Chang TC, Mendell JT, Salzberg SL (2015) StringTie enables improved reconstruction of a transcriptome from RNA-seq reads. *Nature Biotechnol* 33:290-295.

11. Quinlan AR, Hall IM (2010) Genome analysis BEDTools: A flexible suite of utilities for comparing genomic features. *Bioinformatics* 26:841-842.

12. Flicek P, Amode MR, Barrell D, Beal K, Billis K, Brent S, Carvalho-Silva S, Clapham P, Coates G, Fitzgerald S, *et al.* (2014) Ensembl 2014. *Nucleic Acids Res.* 42:749-755.

13. Anders S, Pyl PT, Huber W (2015) HTSeq--a Python framework to work with high-throughput sequencing data. *Bioinformatics* 31(2):166-169.

14. Robinson MD, McCarthy DJ, Smyth GK (2010) edgeR: a Bioconductor package for differential expression analysis of digital gene expression data. *Bioinformatics* 26(1):139-140.

15. Bejamini Y, Hochberg Y (1995) Controlling the false discovery rate: A practical and powerful approach to multiple testing. *J. R. Stat. Soc. B* 57(1):289-300.

16. Altschul SF, Gish W, Miller W, Myers EW, Lipman DJ (1990) Basic local alignment search tool. *J. Mol. Biol.* 215:403-410.

17. Eden E, Navon R, Steinfeld I, Lipson D, Yakhini Z (2009) Gorilla: A tool for discovery and visualization of enriched GO terms in ranked gene lists. *BMC Bioinformatics* 10:48.

18. Morris J, Darolti I, Bloch NI, Wright AE, Mank JE (2018) Shared and Species-Specific Patterns of Nascent Y Chromosome Evolution in Two Guppy Species. *Genes* 9(5):238.
19. Rahman A, Hallgrimsdottir I, Eisen M, Pachter L (2018) Association mapping from sequencing reads using k-mers. *Elife* 7.
20. Quinn A, Juneja P, Jiggins FM (2014) Estimates of allele-specific expression in Drosophila with a single genome sequence and RNA-seq data. *Bioinformatics* 30:2603-2610.
21. Zimmer F, Harrison PW, Dessimoz C, Mank JE (2016) Compensation of Dosage-Sensitive Genes on the Chicken Z Chromosome. *Genome Biol and Evol* 8(4):1233-1242.
22. Koboldt DC, Zhang Q, Larson DE, Shen D, McLellan MD, Lin L, Miller CA, Mardis ER, Ding L, Wilson RK (2012) VarScan 2: Somatic mutation and copy number alteration discovery in cancer by exome sequencing. *Genome Res.* 22:568-576.
23. Stevenson KR, Coolon JD, Wittkopp PJ (2013) Sources of bias in measures of allele-specific expression derived from RNA-seq data aligned to a single reference genome. *BMC Genomics* 14:536.
